# Ethnomedicinal plants used for snakebite treatments in Ethiopia: a comprehensive overview

**DOI:** 10.1590/1678-9199-JVATITD-2019-0017

**Published:** 2019-08-05

**Authors:** Abraham Yirgu, Jean-Philippe Chippaux

**Affiliations:** 1 Central Ethiopia Environment and Forest Research Center, Addis Ababa, Ethiopia.; 2 MERIT, IRD, Paris Descartes University, Sorbonne Paris Cité, Paris, France.; 3 Centre de Recherche Translationnelle, Institut Pasteur, Paris, France.

**Keywords:** Ethnobotany, Medicinal plant, Traditional treatment, Snakebite, Envenomation, Sub-Saharan Africa, Ethiopia

## Abstract

Traditional medicine plays an important role in the daily lives of people living in rural parts of Ethiopia. Despite the fact that Ethiopia has a long history of using traditional medicinal plants as an alternative medicine source, there is no checklist compiling these plants used for snakebite treatment. This review collected and compiled available knowledge on and practical usage of such plants in the country. A literature review on medicinal plants used to treat snakebites was conducted from 67 journal articles, PhD dissertation and MSc theses available online. Data that summarize scientific and folk names, administration methods, plant portion used for treatment and method of preparation of recipes were organized and analyzed based on citation frequency. The summarized results revealed the presence of 184 plant species distributed among 67 families that were cited for treating snakebite in Ethiopia. In this literature search, no single study was entirely dedicated to the study of traditional medicinal plants used for the treatment of snakebite in Ethiopia. Most of the species listed as a snakebite remedy were shrubs and climbers (44%) followed by herbs (33%) and trees (23%). Fabaceae was the most predominant family with the greatest number of species, followed by Solanaceae and Vitaceae. Remedies are mainly prepared from roots and leaves, through decoctions, infusions, powders and juices. Most remedies were administered orally (69%). The six most frequently mentioned therapeutically important plants were *Nicotiana tabacum*, *Solanum incanum*, *Carissa spinanrum*, *Calpurnia aurea*, *Croton macrostachyus* and *Cynodon dactylon*. Authors reviewed the vegetal substances involved in snakebite management and their action mode. In addition to screening the biologically active ingredients and pharmacological activities of these plant materials, future studies are needed to emphasize the conservation and cultivation of important medicinal plants of the country.

## Background

Snakebite is a major public health issue, particularly in sub-Saharan Africa (SSA) [1, 2]. More than 95% of envenomations occur in rural areas, involving persons in agricultural and pastoral work, and leading to many deaths and disabilities [3]. Most epidemiological studies highlight the strong underestimation of incidence and mortality largely because of the complex treatment-seeking behavior of the patients [3, 4]. Snakebite management combines symptomatic and etiological treatment with antivenom [5]. Access to antivenoms is limited in low-income countries for many reasons, including the cost of products and poverty of the majority of patients [6]. Consequently, the population is turning to traditional medicine [7-9] which is a major cause of treatment delay, in addition to poor accessibility of health centers [3, 6].

**Figure 1.**
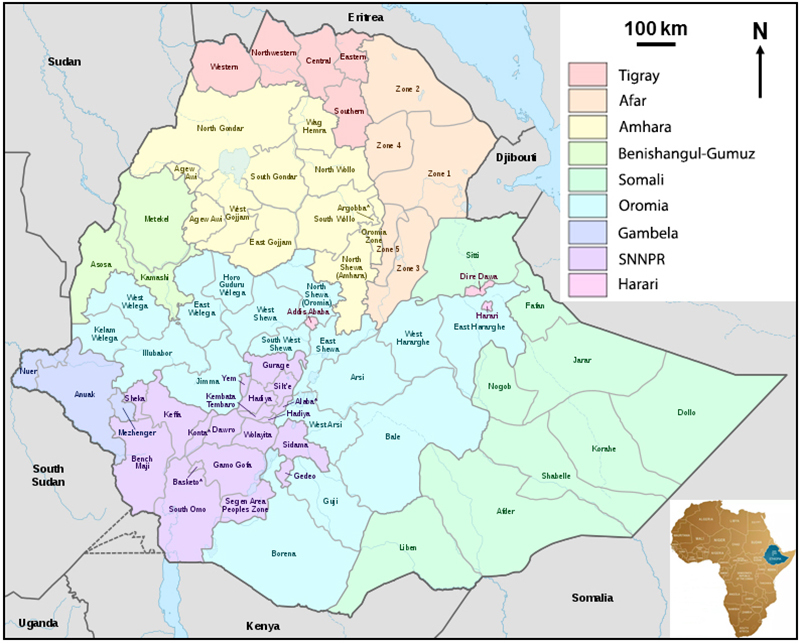
Map showing the geographical location of Ethiopia (modified from https://commons.wikimedia.org/wiki/File:Map_of_zones_of_Ethiopia.svg). SNNPR: Southern Nations Nationalities and People’s Region.

Ethiopia is located in the horn of Africa bordering Eritrea, Djibouti, Somalia, Kenya, South Sudan and Republic of Sudan (Figure 1). According to the United Nations, the population of Ethiopia is estimated at 108 million [10], and comprised of over 80 different ethnic groups. More than 80% of the population reside in rural areas depending on agriculture resources. 

There are more than 98 species of snakes, of which 22 are venomous. Most of them are found at altitudes ranging from 500 to 1,000 m above sea level and are dominated by savanna and desert species [11]. As in most SSA countries, snakebite incidence in Ethiopia is not known accurately due to the lack of case reporting and specific epidemiological studies [3]. However, 949 cases of snakebites were reported in approximately one year from 76 (1%) health facilities in the country [12]. They were able to assess areas of highest incidence particularly in the Oromia, Somali, and Tigray regions. Demographic characteristics were similar to those reported in SSA countries [3]. In these regions, the sex ratio was 2 to 3 (men to women). The population at risk consisted mainly of farmers aged 16-45. The authors noted the lack of antivenom and appropriate documentation in most centers to address this problem. The few published clinical studies [13, 14] have reported high case fatality rates (15-25%) in the absence of appropriate treatment. Hemorrhaging and necrosis were common and presentation delay exceeded 12 hours in most cases. Similar to most SSA countries, most victims attended traditional healers and came to health centers when the symptoms worsened. 

Interestingly, research studies on medicinal plants and traditional ethnomedicine are highly advanced in Ethiopia. Therefore, the main objective of this study is to review information on the traditional medicinal plants employed to treat snakebites in Ethiopia. This study aims to identify plants that would enhance or improve the treatment of envenomations in rural health centers based on properties that have been described in experimental studies. In addition, a comparison with the observations made in other countries would make it possible to identify the plants that are eligible for snakebite treatment [15]. 

## Techniques for data collection

The authors reviewed scientific papers and theses from Medline, Science Digest, Google Scholar and Access to Global Online Research in Agriculture (AGORA) websites using keywords ‘traditional medicinal plants’, ‘snakebite’ and ‘Ethiopia’. Available literature studies were selected based on: (i) reports written in English or French, (ii) articles for which the full texts were available, (iii) publications that present first-hand ethnobotanical information, and (iv) plants with full scientific name (describing the species names as well). A second selection of the first set of documents has been made on the basis of literature reports addressing snakebite in Ethiopia.

All relevant data on each plant species were noted: habit, part(s) used, modes of preparation and administration, and miscellaneous additive comments on the plant utilizations. In the case of discrepancies of plant habit between two reports, the higher growth form of the species was selected and respectively categorized. This was particularly the case when the difference was between “grass” and “shrub” (habit “shrub” was preferred) or between “shrub” and “tree” (we retained tree), etc. In the absence of growth habit, species authority or family names, the Flora of Ethiopia, Global Plants on JSTOR, a digital library [16], Kyalangalilwa et al. [17], Flora of Zimbabwe [18] and the plant list [19] were consulted. Finally, each dataset was prepared in an Excel^®^ spreadsheet with botanical and family names, growth form or habit, plant part(s) used, mode of preparation, route of administration and miscellaneous information. Analysis was performed using Excel^®^ and maps were adapted from Wikipedia and Creative Commons [20]. 

The authors found 67 papers, articles, theses or dissertations on the use of plants in traditional human or veterinary medicine in Ethiopia that have been mentioned for the treatment of snakebites.

**Figure 2.**
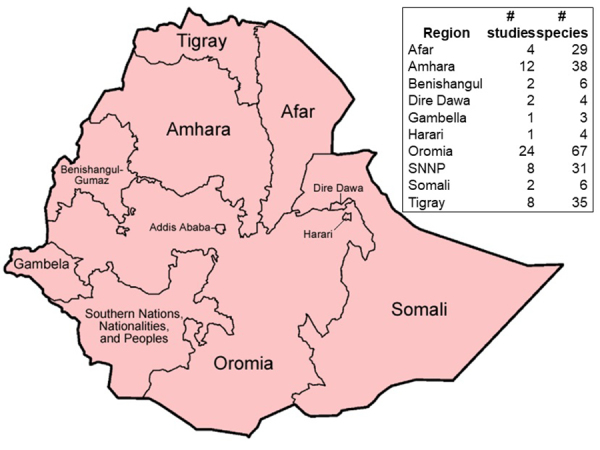
Regional distribution in Ethiopia of studies and ethnomedicinal plants used against snakebites (modified after https://commons.wikimedia.org/wiki/File:Map_of_zones_of_Ethiopia.svg).

Studies were carried out in all regions except in the area administered by the city of Addis Ababa. Most studies were carried out in Oromia, Tigray, Amhara, Afar and Southern Nations Nationalities and People’s Region (SNNPR) regional states (Figure 2).

## Major families of traditional medicinal plants used against snakebites

**Figure 3.**
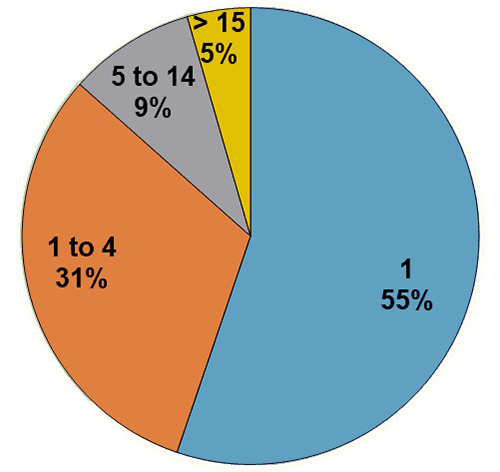
Distribution of ethnomedicinal plant species according to family.

**Figure 4.**
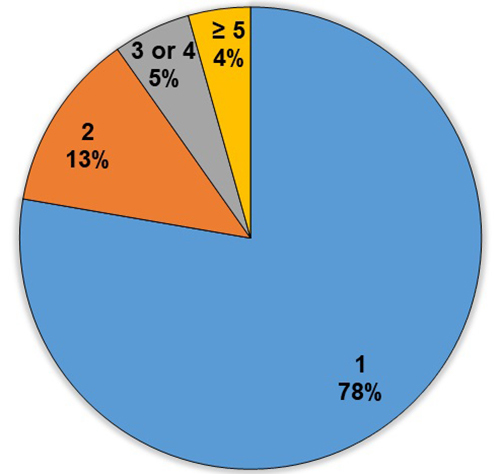
Number of ethnomedicinal plant species mentioned in selected literature references.

**Figure 5.**
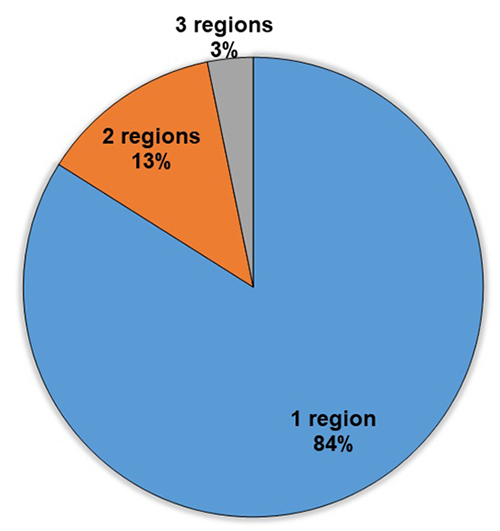
Number of regions sharing each ethnomedicinal plant in Ethiopia.

From 295 citations of plants used for the treatment of snakebites, 184 species were identified in 67 families (Additional file 1). The distribution of the number of species per family showed that three of them had at least 15 species used against snakebites: Fabaceae, Solanaceae and Vitaceae with 29, 22 and 15 species, respectively. Six families had 5 to 14 species and 58 fewer than five species (Figure 3). Most species were listed once (143 i.e., 78%) or twice (23 or 13%), while 18 (9%) were cited more than twice (Figure 4, Table 1). Most species were used against snakebites in only one region of Ethiopia (144 species, 84%). However, eight species (3%) were used in three distinct regions (Figure 5, Table 2). The proportion of plant species employed to prevent or treat snakebites ranged from 1 to 12% in most studies (Table 3).

**Table 1.** List of the most cited ethnomedicinal plant species in Ethiopian studies.

Species	Number of citations	References
*Nicotiana tabacum*	6	[48], [50], [52], [126], [132], [139]
*Solanum incanum*	9	[47], [90], [108],[119], [120], [131], [132], [143], [144]
*Carissa spinarum*	7	[46], [90], [93], [109], [110], [112], [118]
*Calpurnia aurea*	6	[52], [91], [103], [120], [134], [135]
*Croton macrostachyus*	6	[46], [107], [121], [123], [129], [130]
*Cynodon dactylon*	6	[46], [107], [120], [131], [132], [134]
*Cyphostemma adenocaule*	4	[50], [98], [99], [140]
*Cyphostemma junceum*	5	[54], [98], [99], [117], [118]
*Plumbago zeylanica*	4	[93], [109], [117], [118]
*Polygala abyssinica*	4	[50], [90], [98], [102]
*Verbena officinalis*	4	[50], [104], [117], [118[]
*Commiphora myrrha*	2	[125], [126]
*Echidnopsis dammaniana*	3	[97], [115], [116]
*Euclea racemosa*	3	[97], [98], [99]
*Gossypium herbaceum*	3	[50], [117], [118]
*Seddera hirsuta*	2	[94], [95]
*Stereospermum kunthianum*	3	[53], [107], [120]
*Vernonia adoensis*	3	[54], [107], [123]

**Table 2.** List and regional distribution of ethnomedicinal plant species used for snakebite treatment present in at least three regions of Ethiopia.

Species	Amhara	Somali	Oromia	Tigray	Afar	SNNPR	Harari	Gambella	Benishangul
*Carissa spinarum*	X	X	X	X	X				
*Solanum incanum*		X	X	X	X	X			
*Calpurinia aurea*			X		X	X	X		
*Croton acrostachyus*	X		X	X					X
*Cucumis dipsaceus*			X	X	X				X
*Cucumis ficifolius*			X	X	X	X			
*Nicotiana tabacum*			X	X	X	X			
*Plumbago zeylanica*	X		X	X	X				
*Silene macrosolen*	X			X	X		X		
*Cynodon dactylon*			X	X		X			
*Cyphostema adenocaule*			X	X	X				
*Echidnopsis dammaniana*			X	X	X				
*Euclea racemosa*			X	X	X				
*Jasminum abyssinicum*	X				X	X			
*Leonotis ocymifolia*	X		X		X				
*Olea europaea*	X		X		X				
*Rhus natalensis*	X		X		X				
*Senna obtusifolia*			X		X	X			
*Stereospermum kunthianum*	X		X		X				
*Verbena officinalis*			X	X	X				
*Withania somnifera*			X		X			X	

**Table 3.** Number of ethnomedicinal plant species used against snakebites (preventive and curative) in Ethiopia and some sub-Saharan African countries.

Country	Number of families	Number of Species	Used for snakebites	References
Ethiopia	57	122	2 (2%)	[111]
Ethiopia	31	49	0	[146]
Ethiopia	23	47	2 (4%)	[137]
Ethiopia	49	82	3 (4%)	[134]
Ethiopia	46	75	4 (5%)	[120]
Ethiopia	51	93	?	[147]
Ethiopia	33	38	2 (5%)	[129]
Ethiopia	33	53	3 (6%)	[40]
Ethiopia	31	62	6 (10%)	[133]
Ethiopia	47	120	5 (4%)	[91]
Ethiopia	29	43	1 (2%)	[122]
Ethiopia	40	83	4 (5%)	[97]
Ethiopia	28	51	3 (6%)	[100]
Ethiopia	44	85	2 (2%)	[143]
Ethiopia	23	34	0	[148]
Ethiopia	23	42	1 (2%)	[125]
Ethiopia	43	81	3 (4%)	[139]
Ethiopia	64	135	7 (5%)	[93]
Ethiopia	67	163	10 (6%)	[107]
Ethiopia	51	87	10 (11%)	[132]
Ethiopia	65	155	11 (7%)	[90]
Ethiopia	46	106	5 (5%)	[105]
Ethiopia	46	106	3 (3%)	[106]
Ethiopia	34	49	4 (8%)	[119]
Ethiopia	21	49	4 (8%)	[115]
Ethiopia	34	67	3 (4%)	[128]
Ethiopia	46	83	4 (5%)	[96]
Ethiopia	28	32	2 (6%)	[130]
Ethiopia	23	33	3 (9%)	[47]
Ethiopia	23	49	1 (2%)	[92]
Ethiopia	48	76	1 (1%)	[124]
Ethiopia	34	35	0	[149]
Ethiopia	27	51	2 (4%)	[121]
Ethiopia	26	34	0	[150]
Ethiopia	47	115	12 (10%)	[53]
Ethiopia	27	34	1 (3%)	[127]
Ethiopia	20	30	1 (3%)	[116]
Ethiopia	54	131	4 (3%)	[140]
Ethiopia	26	42	0	[151]
Ethiopia	49	128	7 (5%)	[109]
Ethiopia	68	213	3 (1%)	[126]
Ethiopia	50	85	6 (7%)	[48]
Ethiopia	62	147	6 (4%)	[131]
Ethiopia	48	54	2 (4%)	[141]
Ethiopia	74	230	0	[152]
Ethiopia	71	135	1	[103]
Ethiopia	35	51	4 (8%)	[114]
Ethiopia	59	145	6 (4%)	[46]
Ethiopia	56	126	5 (4%)	[52]
Ethiopia	46	113	0	[14]
Ethiopia	58	101	5 (5%)	[54]
Ethiopia	29	60	7 (12%)	[123]
Ethiopia	18	31	1 (3%)	[101]
Ethiopia	28	58	2 (3%)	[102]
Ethiopia	57	133	3 (2%)	[112]
Ethiopia	39	72	3 (4%)	[49]
Ethiopia	27	60	2 (3%)	[144]
Ethiopia	18	70	14 (20%)	[94]
Ethiopia	53	114	10 (9%)	[98]
Ethiopia	27	50	6 (12%)	[99]
Ethiopia	40	60	3 (5%)	[110]
Ethiopia	42	67	5 (7%)	[118]
Ethiopia	51	80	6 (8%)	[113]
Ethiopia	40	57	2 (4%)	[138]
Ethiopia	33	91	10 (11%)	[104]
Ethiopia	46	71	0	[153]
Ethiopia	41	83	2 (2%)	[136]
Ethiopia	37	74	0	[154]
Ethiopia	?	27	1 (4%)	[145]
Ethiopia	17	24	1 (4%)	[142]
Ethiopia	44	68	3 (4%)	[51]
Ethiopia	23	26	2 (8%)	[135]
Benin	69	114	0	[155]
Cameroon	26	39	2 (5%)	[29]
Chad	19	38	4 (11%)	[156]
Djibouti	40	91	6 (7%)	[27]
Eritrea	?	256	15 (6%)	[157]
Eritrea	27	55	0	[158]
Kenya	26	48	0	[159]
Nigeria	95	325	9 (3%)	[26]
Soudan	31	53	2 (4%)	[28]
South Africa	42	82	2 (2%)	[160]

**Figure 6.**
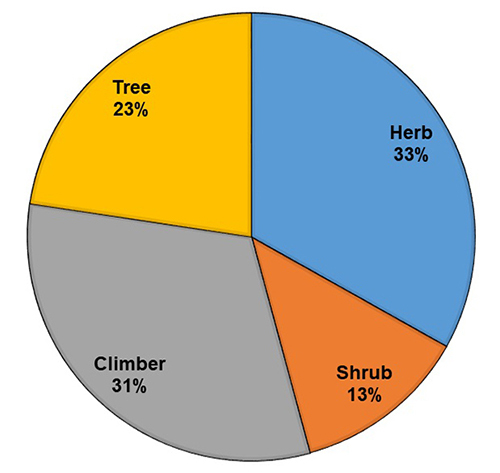
Distribution of ethnomedicinal plant habits used against snakebite in Ethiopia.

**Figure 7.**
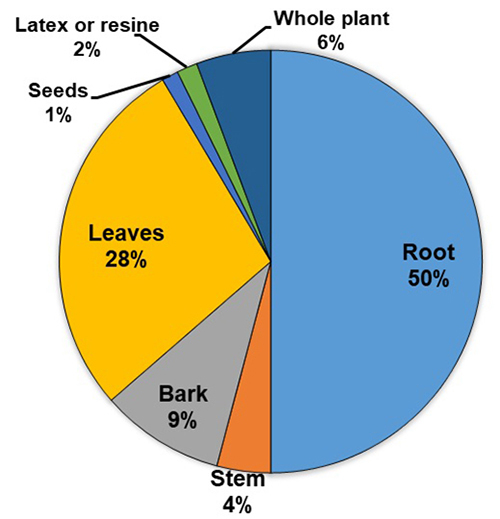
Ethnomedicinal plant parts used against snakebite in Ethiopia.

**Figure 8.**
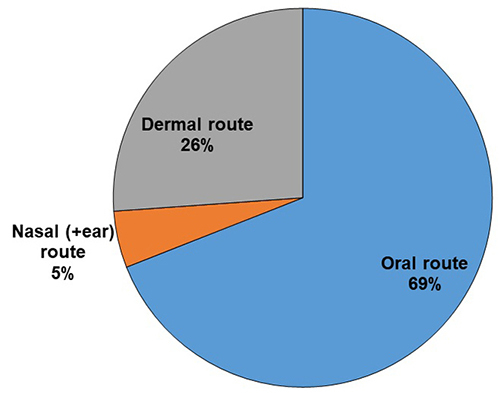
Administration routes used for ethnomedicinal plants used against snakebite in Ethiopia.

Figure 6 shows the distribution of plants according to their habit. The majority of species were small (herb, shrub or climber), while 23% were trees. In half of the cases, the roots were used alone or in combination with another part of the plant. Leaves accounted for more than a quarter of uses while other parts of the plant accounted for less than 10% each, most often in association with other parts (Figure 7). The mode of preparation varied according to the plant, authors, traditional healers and regions. The oral route was most frequently used followed by local application (Figure 8). 

Medicinal plants are an alternative form of medication utilized by most rural communities around the world. They largely rely on the support of traditional healers that prescribe these medicinal plants for various ailments [21-24]. In addition to large-size molecules (fatty acids, glucosides and peptides) acting as energy reserves or metabolic precursors, low-molecular-weight compounds protect plants from their environment. They present intense and polyvalent biological activities resulting in various pharmacological effects [25]. In addition, a reduction in snakebite risk is achieved by repulsing snakes either by fumigation or by growing repellent plants around houses, such as *Nicotiana tabacum* and *Datura metel* [26]. 

Several traditional medicinal plant families were reported to be efficacious at treating or avoiding snakebite in different countries. Some of these included: Aloaceae, Aristolochiaceae, Fabaceae, Lamiaceae and Rhamnaceae in Djibouti [27], Caesalpiniaceae and Mimosaceae in Sudan [28], Acanthaceae, Amaranthaceae, Asclepiadaceae, Burseraceae, Commelinaceae, Lamiaceae and Loganiaceae in Nigeria [26], and Melastomataceae and Menispermaceae in Cameroon [29]. The most commonly encountered families listed were: Acanthaceae, Amaranthaceae, Amaryllidaceae, Compositae, Celastraceae, Capparaceae, Burseraceae, Brassicaceae, Boraginaceae, Aristolochiaceae, Araceae, and Apocynaceae [30]. Acanthaceae, Amaranthaceae, Apocynaceae, Araceae, Asteraceae, Caesalpiniaceae, Cucurbitaceae, Euphorbiaceae, Fabaceae, Lameaceae, Moraceae, Rubiaceae, Rutaceae and Zingiberaceae were mentioned in India [31] as important families of medicinal plants for snakebites or snake repellent, whereas families such as Apocynaceae, Araceae, Aristolochiaceae, Asteraceae, Convolvulaceae, Fabaceae, Passifloraceae, Piperaceae, Polygonaceae, Rubiaceae and Solanaceae were reported in Central America [32]. Two families commonly recognized for their medicinal values in Ethiopia were described in other countries: Fabaceae in Djibouti, India and Central America; and Lamiaceae in Djibouti, Nigeria and India.

In SSA, snakebite patients primarily consult traditional healers [3]. Several reasons explain this treatment-seeking behavior [3, 4, 8]. The main causes of delay until hospital presentation or rejection of modern medicine have been highlighted: (i) popular beliefs, (ii) impression of relative effectiveness of traditional treatments due to the fact that the majority of bites are not fatal and that many of them are not followed by envenomation, (iii) greater logistical and financial accessibility of traditional medicine, and (iv) inadequate management of snakebites in health centers due to a lack of drugs or properly trained staff [3, 4, 6, 8, 33]. 

In Ethiopia, 184 plant species from 67 families (more than a third belonging to Fabaceae, Solanaceae or Vitaceae) have been reported. The comparison between Ethiopian studies and those in other SSA countries is displayed in Table 3 just as an indication because the search for studies performed in countries other than Ethiopia was not conducted with the same objective of completeness. The high proportion of plants used against snakes and snakebites is probably due to the high incidence of snakebites [1, 2] and by the position of snakes in traditional cultures [34]. 

## Factors that determine the choice of traditional medicinal plants

The choice of the plants by traditional healers depends on complex mechanisms [35]. The smell and appearance of the plant, such as the presence of aerial roots reminiscent of the snake's body [36-38], folklore legends [39], observation and empiricism that support the curative action of plants [36] have led traditional medicine to preferentially use some of medicinal plants to treat snakebites. Information circulates mainly vertically and endogenously. Training is shared within the community between experienced traditional healers and their students, who are chosen mainly among male children within their own family [40]. Comparison of the use of plants against snakebites in two distinct ethnic groups in Kenya showed that the choice depended on independent cultural origins [41]. This could explain why few plant species are used against snakebites simultaneously in several regions (Figure 5, Table 2). Another reason could be that analog active substances are widely distributed among species of the same family, or even close families, which constitutes a redundancy by allowing a use of different plants for the same disease, or of the same plant for distinct ailments, considering symptomatic treatments.

For example, *Securidaca longepedunculata* (Polygalaceae) is one of the main herbs used as an anti-inflammatory, analgesic and specific antidote for snakebites in many West African communities [35, 36, 42-45]. In Ethiopia, the species is widely traded [46] due to environmental constraints, deforestation and other uses that endanger it [47-50]. In other parts of SSA, *S. longepedunculata* is used against evil eye and/or as a psychotropic [28, 45, 51-55]. However, although *S. longepedunculata* is frequently used in Ethiopia as anti-inflammatory or analgesic [46, 48, 51], it is likely that traditional practitioners do not utilize this plant for symptomatic treatment of snakebite. As a result, it may be interesting to search for plants used against snakebites in several communities, assuming that this convergence reflects widely recognized efficacy and, secondly, identify plants belonging to the same family that contain substances of interest [15]. 

In traditional medicine, the use of plants is strongly linked to spiritual and religious practices associating the administration of the plant or its extract with incantations, offerings, ritual gestures or prayers [36]. These practices are mentioned in several studies of our series but never detailed. Their psychosomatic role is nevertheless considered essential, especially in relation to the anxiety of the patient and his entourage, and an important asset for healing. 

## Plant parts, preparation methods and application mode of remedies

Roots and leaves are used in three quarters of snakebite preparations. On the other hand, plant embryonic forms, such as the seed, bud or bulb (gemmotherapy) which concentrate certain substances of the plant [36], remain exceptionally prescribed, e.g. six times in our series (Additional file 1).

Plant preparation is an important aspect. The extraction of active substances depends on the part of the plant or the solvent used, and influences the efficacy and toxicity. The aqueous extract of *Nicotiana rustica* (Solanaceae) had no significant effect on the proteases of *Naja nigricollis* (Elapidae) venom whereas the ethanolic extract completely inhibits its proteolytic activity [56]. We have collected little precise information on the preparation of plants or extracts before their administration. In Ethiopia, aqueous extractions are the most common. Electuaries made with butter or honey are sometimes mentioned, but alcoholic solutions are uncommon. With regard to a medical emergency such as snakebite, many traditional healers prefer ready-to-use stable preparations such as powders, paste or dried plants [36]. 

External uses represented one-third of uses. Internal uses were mainly achieved by oral administration (70%). Endermic routes, obtained by rubbing the plant on the skin or by contact with a mucous membrane, particularly the mouth or the nose to allow rapid diffusion, were observed in nearly 10%. The intra-rectal route (enema), which produces results similar to parenteral injection of most micromolecules, was not mentioned in our series. The dosage is rarely specified because it is not a factor considered by traditional healers as decisive, apart from mystical considerations [36, 38, 57]. 

## Health effects of snakebite and chemical composition of venom

Snakebites cause an inflammatory syndrome associating pain, edema, hemorrhaging and necrosis by enzymatic digestion of the tissues and lack of vascularization, or neuromuscular paralysis by blocking nerve impulse [58]. These symptoms are often complicated by failure of major organs (heart, lung, kidney, brain, etc.). Snake venoms are essentially composed of low-molecular-weight toxins acting on a membrane receptor, and enzymes that hydrolyze various types of molecules. African Viperidae venoms are particularly rich in enzymes responsible for inflammation, hemorrhaging and necrosis. Serine proteases (SP) activate coagulation and lead to the consumption of coagulation factors until they are depleted, resulting in bleedings. Hyaluronidase promotes the spreading of venom in the body. Zn-Metalloproteinases (MP) hydrolyze vascular endothelium causing extravasation of blood from the vessels. L-amino acid oxidase (L-AAO) is responsible for apoptosis and cytotoxicity, and acts on platelet aggregation. Phospholipases A_2_ (PLA_2_s), according to their structure, are responsible for several toxic actions: (i) activation of factor X of blood coagulation in which the calcium ions intervene, causing consumption of coagulation factors such as SP, (ii) hydrolysis of platelet membrane, preventing blood clot formation, and iii) activation of inflammation mediators.

African Elapidae have venoms composed of (i) cytotoxins (CT), (ii) neurotoxins (NT), and (iii) enzymes, particularly PLA_2_s. CT target membrane receptors, destroying the cell, whereas NT block cholinergic receptors, causing paralysis of respiratory muscles. PLA_2_s activate inflammation mediators and destroy muscles, leading to paralysis and necrosis.

Plants present two modes of action: symptomatic effects and antidotes [35]. Symptomatic treatment aims at alleviating or eliminating pain or edema, as well as hemorrhaging and necrosis. This action results from indirect intervention in the pathologic mechanisms. In contrast, antidotes inhibit venom action. Plants can also act preventively against the deleterious effects of venom when the plant is administered prior to the bite [25]. 

Generally, the efficacy of plant extracts is measured *in vivo* by their capacity to neutralize the lethal dose 50% (LD_50_) of the venom in mice. Some tests are performed *in vivo* or *in vitro* on specific toxic activities (hemorrhaging, necrosis, neurotoxicity, etc.) using models focused on more accurate biological targets [58]. 

## Effects of plant extraction method on the efficacy of plant antivenom mechanism

Many plants present a significant antivenom effect, although the mechanism is still poorly documented. Methanol extract of *Crinum jagus* (Amaryllidaceae), administered orally or intraperitoneally after incubation with the venom or separately, protected mice against an injection of venom from *Echis ocellatus*, *Bitis arietans* (Viperidae) and *Naja nigricolis* [59]. The methanol extract from leaves of *Guiera senegalensis* (Combretaceae) reduced mortality of mice inoculated with venom of *Echis ocellatus* and *Naja nigricollis* [60]. The ethanol extract of *Diodia scandens* (Rubiaceae) reduced the systemic toxicity of *Echis carinatus* venom [61]. Neurotoxic effects of Elapidae venom and hemorrhaging due to Viperidae venom were neutralized by aqueous and methanol extracts of *Parkia biglobosa* (Mimosaceae) [62]. However, the results were not confirmed *in vivo* in mice whose mortality was not reduced but only delayed. A low neutralization of venoms of *Dendroaspis jamesoni* (Elapidae) and *Echis ocellatus* was observed in mice after oral administration of *Schumanniophyton magnificum* (Rubiaceae), *Bidens pilosa* (Asteraceae), and *Garcinia lucida* (Clusiaceae) [43]. The methanol extract from *Boswellia dalzielli* (Burseraceae) partially neutralized the toxic effect of *Echis ocellatus* venom in previously envenomed rats [63].

Molander et al. [64] studied 226 extracts from 94 SSA plants likely to inhibit the enzymatic activities (hyaluronidase, PLA_2_ and proteases) of *Bitis arietans* and *Naja nigricollis* venoms. Fabaceae, Anacardiaceae and Malvaceae contain the largest number of species that neutralize snake venom enzymes. The aqueous extracts of *Pupalia lappacea* (Amaranthaceae), *Combretum molle* (Combretaceae), *Strychnos innocua* (Laganiaceae) and *Grewia mollis* (Tiliaceae), and ethanol extracts of *Lannea acida* (Anacardiaceae) and *Bauhinia thonningii* (Fabaceae) - even after elimination of polyphenols, whose action is nonspecific - retained an inhibitory activity of hyaluronidase and proteases. The aqueous extract of some Urticaceae, Asteraceae or Rubiaceae inhibited the production of mediators involved in the local and systemic inflammatory process induced by venoms, notably by inhibition of prostaglandin synthesis as effectively as indomethacin or dexamethasone [44, 65-68]. 

PLA_2_ inhibitors have been among the most studied [69]. Many phenolic compounds inhibit PLA_2_s, and include: (i) flavonoids, coumestans, alkaloids and various carboxylic acids such as acetylsalicylic, aristolochic, chlorogenic and caffeic acids, (ii) steroids (sterols and cholesterol) and (iii) terpenoids which include oleanolic acid and lupeol [69, 70]. 

*Schumanniophyton magnificum*, *Eclipta prostrata* (Asteraceae) and *Aristolochia shimadai* (Aristolochiaceae) extracts inhibited PLA_2_ venom activity [71]. The ethanol extract of *Anacardium occidentale* (Anacardiaceae) neutralized the enzymes (PLA_2_, protease and hyaluronidase) from *Daboia russelii* venom (Viperidae) and inhibited venom-induced edema, hemorrhaging, myotoxicity and lethality [72]. The methanol extract of *Leucas asperas* (Lamiaceae) neutralized the proteases and hyaluronidase of the *Naja naja* venom (Elapidae), as well as its hemolytic effects but did not inhibit PLA_2_ activity [73]. Pinostrobin, a flavonoid isolated from the dichloromethane extract of *Renealmia alpinia* (Zingiberaceae), significantly inhibited the enzymatic and hemolytic activities of *Bothrops asper* (Viperidae) and attenuated tissue damage and hemorrhagic effects with a documented peripheral and anti-inflammatory analgesic action [74, 75].

Vegetal substances act by different mechanisms on the venom proteins, leading to partial or total neutralization of their activities. Antivenom may act by different mechanisms to neutralize the venom proteins: (i) precipitation, (ii) structural modification, (iii) alteration of the function, particularly by chelation of the metal ion necessary for the activity, (iv) destruction, (v) competition or antagonism, in particular by steric hindrance, taking its place - or a nearby place - on the receptor of the toxin or the enzyme. 

The inhibition of the hemorrhagic effect of the venom of *Bothrops jararaca* (Viperidae) by certain plants is generally accomplished by inactivation of the venom enzymes without structural degradation, as confirmed by the electrophoresis of the venom proteins after incubation of the plant extract with the venom [76]. Black tea melanin contains a polyphenol that counteracts the toxicity of Viperidae venoms by calcium chelation and nonspecific inhibition of PLA_2_s from venoms [77]. Some organic plants extracts, particularly Euphorbiaceae, inhibit Viperidae MPs responsible for hemorrhaging, probably by chelation of the zinc ion necessary for catalytic activity [76, 78]. 

The aqueous extract of *Aristolochia indica* root (Aristolochiaceae) partially destroyed the proteins of the venom of *Daboia russelii* (Viperidae), probably through the action of aristolochic acid. This alkaloid isolated from *Aristolochia* species is a noncompetitive inhibitor of venom enzymes [79]. The interaction between aristolochic acid and PLA_2_s caused a modification of the secondary structure of the protein without detectable change in its tertiary structure [80, 81]. 

The methanol extract of *Schumanniophyton magnificum* inactivated previously administered venom of *Naja melanoleuca* [82]. Schumanniofoside probably oxidized NT disulfide bridges, which are essential for the toxicity. A similar effect has been observed against cobra venom CT structurally analogous to the NT [83]. 

The protective effect that results from an administration of a substance prior to the envenomation depends on molecular interactions between the plant substance and venom action site. For example, seven very young twigs of *Annona senegalensis* (Annonaceae) and seven seeds of *Aframomum melegueta* (Zingiberaceae) are eaten to avoid snakebites [26]. In addition to the mystical side of the preparation and the symbolic significance of the number 7, the antivenom effect has been demonstrated [84, 85]. The aqueous extract of *Annona senegalensis*, deposited on the nerve-muscle preparation of the gastrocnemius of amphibian before the venom of *Bitis arietans*, showed a significantly higher antivenom effect than the mixture of the extract with the venom, suggesting a preventive rather than curative efficacy. However, the mechanism of this effect remains unclear because *B. arietans* venom is known to be proteolytic and devoid of neurotoxicity, at least in mammals. Similarly, *Securidaca longepedunculata* extract antagonized the paralyzing action of *Naja nigricollis* venom on an isolated amphibian nerve-muscle preparation in a dose-dependent manner [42]. The authors hypothesized that *S. longepedunculata* extract binds the vicinity of the cholinergic receptor without modifying its activity but preventing the paralysis due to NT [42, 43, 58]. 

The aqueous extract of lectin-bearing *Mucuna pruriens* seeds (Fabaceae), inoculated in mice 24 hours before the administration of *Echis carinatus* venom protected the mice, whereas the incubation of the venom with the extract did not exert a neutralizing effect [86]. The lectin has a conserved domain similar to an epitope of venom PLA_2_ that binds to factor X. The lectin binds to factor X by this epitope, preventing PLA_2_ from binding to a coagulation factor. 

However, ELISA and immunotransfer studies have shown cross-reactions between rabbit IgG against plant extract and snake venoms, the neutralization of which was specifically conferred by *M. pruriens* seed extract [87]. The results suggested that some plant proteins have common epitopes with toxins or enzymes of the venom allowing a competition with the latter that prevents their attachment to the receptors. 

Finally, plants can also have an adjuvant effect on the defense mechanisms of an envenomation victim. Proteomic studies showed that aqueous extract of *Mucuna pruriens* (Fabaceae) caused major changes in the plasma proteome inoculated with *Echis carinatus* venom (Viperidae), suggesting that the systemic but nonspecific protective effect against coagulant and inflammatory activities would enable the victim to endure the critical phase of envenomation [88]. The benefits can be an increasing survival time, reduction of toxic signs, improvement of diaphragmatic contraction and inhibition of proteolysis. The cardiovascular system, in particular, is protected by the action of certain plants on blood pressure, atrial contractility and rhythm, or the prevention of endothelial damage [89]. 

## Conclusion

Plants remain the main therapeutic remedy for sub-Saharan populations, particularly in rural areas where snakebites are common. It is therefore essential to inventory them and ensure their effectiveness. In addition, the uses of traditional medicinal plants are well recognized for their contribution to developing new drugs that assist in overcoming public health problems. Accordingly, the authors attempted to compile both published and unpublished literatures available online, although these studies were limited to fewer than 25 percent of the total districts of Ethiopia. In addition, the lowlands that are largely recognized as the biogeography of snakebites are not well addressed. However, they are highly representative of the phytopharmacopeia against snakebite in Ethiopia and could serve as a basis for further studies. Data on medicinal plants and their uses should be gathered through contextualized ethnomedical studies. Besides the need for first-hand information from the abovementioned districts, future studies need to evaluate the phytochemical constituents of most widely used medicinal plants of Ethiopia. In addition, experimental activities of a plant substance must be confirmed and validated by standardized clinical trials in humans.

### Abbreviations

SSA: sub-Saharan Africa; SNNPR: Southern Nations Nationalities and People’s Region; SP: serine proteases; MP: metalloproteinases; L-AAO: L-amino acid oxidase; PLA_2_s: phospholipases A_2_; CT: cytotoxins; NT: neurotoxins; LD_50_: lethal dose 50%.

## Supplementary material

The following online material is available for this article:

Additional file 1. Scientific name, habit, part used, method of preparation, route of administration and applications of ethnomedicinal plants employed in Ethiopia for snakebite treatment.
