# Supplementary material for: Ethnomedicinal plants used for snakebite treatments in Ethiopia: a comprehensive overview
Source: J Venom Anim Toxins Incl Trop Dis. 2019 Aug 5;25:e20190017. doi: 10.1590/1678-9199-JVATITD-2019-0017 (PMC6682375; doi:10.1590/1678-9199-JVATITD-2019-0017)
Supplement: Additional file 1. [file 1678-9199-jvatitd-25-e20190017-s1.pdf]

Supplementary Material to "Ethnomedicinal plants used for snakebite treatments in Ethiopia: a comprehensive overview"

Additional file 1. Scientific name, habit, part used, method of preparation, route of administration and applications of ethnomedicinal plants employed for snakebite treatment in Ethiopia.

Family	Species	Habit	Part used	Preparation	Traditional therapeutic use	Comments	Reference
Acanthaceae	*Barleria eranthemoides*	Shrub	Root	Crushed and mixed with water	Oral route		90
Acanthaceae	*Justicia betonica*	Herb	Root	Crushed and mixed with water	Oral route	Veterinary use	91
Acanthaceae	*Justicia schimperiana*	Shrub	Leaves		Oral route	Veterinary use	92
Acanthaceae	*Ruellia patula*	Herb	Root	Chewed and swallowed	Oral route		93
Aizoaceae	*Zaleya pentandra*	Herb	Leaves	Crushed	Local application		94
Aizoaceae	*Zaleya pentandra*	Herb	Root	Crushed and mixed with water	Nasal route		94
Aizoaceae	*Trianthema portulacastrum*	Herb	Whole plant		Nasal route		95
Alliaceae	*Allium sativum*	Herb	Bulb	Crushed to paste	Local application		96
Aloaceae	*Aloe harlana*	Shrub	Leaves	Crushed and mixed with water	Oral route		97
Aloaceae	*Aloe megalacantha*	Shrub	Whole plant	Crushed to juice	Oral route		98
Aloaceae	*Aloe megalacantha*	Shrub	Whole plant	Crushed to juice; chewed and swallowed	Oral route		99
Aloaceae	*Aloe pirottae*	Shrub	Leaves	Decoction	Oral route	Associated with other ingredients	100
Aloaceae	*Aloe* spp.	Shrub	Root			Veterinary use	101
Aloaceae	*Aloe trichosantha*	Shrub	Root		Oral and nasal routes; body wash		95
Amaranthaceae	*Achyrantes aspera*	Herb	Root	Chewed and swallowed	Oral route		98
Amaranthaceae	*Achyrantes aspera*	Herb	Root	Chewed and swallowed	Oral route		99
Amaranthaceae	*Aerva javanica*	Shrub	Root	Burned to charcoal and mixed with butter	Local application		94
Amaranthaceae	*Aerva javanica*	Shrub	Root	Chewed and swallowed	Oral route		102
Amaranthaceae	*Cyatula cylindrica*	Herb					103
Amaryllidaceae	*Crinum abyssinicum*	Herb	Root	Crushed	Local application		54
Anacardiaceae	*Operculicarya gummifera*	Tree			Oral route	Also veterinary use	104
Anacardiaceae	*Rhus natalensis*	Shrub	Leaves	Chewed and swallowed	Oral route		105; 106
Anacardiaceae	*Rhus natalensis*	Shrub	Leaves		Oral route; local application		95
Apiaceae	*Heteromorpha arborescens*	Shrub	Root	Chewed and swallowed	Oral route; application on the neck		107
Apocynaceae	*Acokanthera schimperi*	Tree	Whole plant	Fumigation		Used as snake killer	57
Apocynaceae	*Acokanthera schimperi*	Tree	Leaves				51
Apocynaceae	*Carissa edulis*	Shrub	Root	Infusion	Oral route	Veterinary use	108
Apocynaceae	*Carissa spinarum*	Tree	Root	Chewed and swallowed	Oral route		90

**Family**	**Species**	**Habit**	**Part used**	**Preparation**	**Traditional therapeutic use**	**Comments**	**Reference**
Apocynaceae	*Carissa spinarum*	Tree	Leaves	Fumigation		Use as snake repellent	109
Apocynaceae	*Carissa spinarum*	Tree	Root		Oral route		46
Apocynaceae	*Carissa spinarum*	Tree	Root	Powedered and mixed with water	Poured in snake's hole	Used as repellent	110
Apocynaceae	*Calotropis procera*	Tree	Root	Chewed and swallowed	Oral route		111
Apocynaceae	*Calotropis procera*	Tree	Root; leaves	Crushed to juice	Oral route; local application		53
Apocynaceae	*Carissa carandas*	Shrub	Leaves	Chopped and mixed with water	Oral route		104
Apocynaceae	*Carissa spinarum*	Tree	Leaves	Chewed and swallowed	Oral route		93
Apocynaceae	*Carissa spinarum*	Tree	Leaves	Chewed and swallowed	Oral route		112
Apocynaceae	*Carissa spinarum*	Tree	Root	Chewed and swallowed	Oral route		113
Apocynaceae	*Dregea schimperi*	Climber	Root	Crushed and mixed with water	Oral route	Veterinary use	114
Apocynaceae	*Echidnopsis dammaniana*	Herb	Stem	Crushed	Local application		97
Apocynaceae	*Echidnopsis dammaniana*	Shrub	Stem	Crushed and mixed with water; crushed to paste	Local application	Veterinary use	115
Apocynaceae	*Echidnopsis dammaniana*	Shrub	Leaves	Crushed and mixed with water	Oral route; local application		116
Apocynaceae	*Pergularia daemia*	Climber	Root		Local application		117
Apocynaceae	*Pergularia tomentosa*	Herb			Oral, nasal and ear routes		95
Apocynaceae	*Sarcostemma andongense*	Herb	Leaves	Leaf juice	Oral route	Veterinary use	115
Araliaceae	*Schefflera abyssinica*	Tree	Stem; bark	Water infusion	Oral route		93
Aristolochiaceae	*Aristolochia bracteolata*	Climber	Root		Oral route		95
Asclepiadaceae	*Pergularia daemia*	Climber	Root		Local application	Applied after wound incisions	118
Asparagaceae	*Albuca abyssinica*	Herb	Root	Crushed and mixed with water	Oral route	Veterinary use	119
Asteraceae	*Echinops kerebicho*	Herb		Fumigation		Use as repellent	40
Asteraceae	*Vernonia adoensis*	Shrub	Root	Decoction	Oral route	Associated with *Catha edulis*	54
Asteraceae	*Vernonia bipontini*	Shrub	Root	Crushed and filtered	Oral route		98
Asteraceae	*Cirsium englerianum*	Herb		Crushed and mixed with water	Oral route		49
Asteraceae	*Echinops kerebicho*	Herb	Root	Fumigation		Used as repellent	120
Asteraceae	*Galinsoga quadriradiata*	Herb	Leaves		Local application		121
Asteraceae	*Guizotia scabra*	Shrub	Root	Crushed and mixed with water	Oral route	Associated with *Acacia brevispica* and *Ficus vasta*	48
Asteraceae	*Gymnanthemum amygdalinum*	Tree	Root	Chewed and swallowed	Oral route		50
Asteraceae	*Gymnanthemum auriculiferum*	Shrub		Crushed and mixed with water	Oral route		122
Asteraceae	*Kleinia odora*	Shrub	Whole plant	Fumigation		Used as repellent	50

Family	Species	Habit	Part used	Preparation	Traditional therapeutic use	Comments	Reference
Asteraceae	*Vernonia adoensis*	Shrub	Root	Crushed and mixed with water or chewed and swallowed	Oral route		107
Asteraceae	*Vernonia adoensis*	Shrub	Root	Crushed and mixed with water	Oral route		123
Asteraceae	*Vernonia auriculifera*	Shrub	Root	Crushed and mixed with water	Oral route	Also veterinary use	49
Bignoniaceae	*Stereospermum kunthianum*	Tree	Stem; bark	Chewed and swallowed	Oral route; local application	Use for snakebite and scorpion sting	93
Bignoniaceae	*Stereospermum kunthianum*	Tree	Root; bark		Oral route; local application	Use for snakebite and scorpion sting	124
Bignoniaceae	*Stereospermum kunthianum*	Tree	Root	Root powder mixed with water and swallowed	Oral route	Veterinary use; associated with *Calpurnia aurea*	120
Bignoniaceae	*Tecomaria capensis*	Tree	Root	Crushed and mixed with water	Oral and nasal route		53
Boraginaceae	*Helotropium rariflorum*	Herb	Whole plant	Crushed and mixed with water	Body wash	Associated with unidentified plant	94
Burseraceae	*Boswellia papyrifera*	Tree	Root; latex	Fumigation		Associated with *Calotropis procera*; also veterinary use; snake repellent	53
Burseraceae	*Commiphora myrrha*	Tree	Stem	Crushed	Oral route; local application	Anti-hemorrhagic and healing effects; used as snake repellent	125
Burseraceae	*Commiphora myrrha*	Tree	Resine	Chewed and swallowed	Oral route		126
Burseraceae	*Commiphora myrrha*	Tree	Whole plant	Fumigation		Used as repellent	126
Cactaceae	*Opuntia ficus-indica*	Shrub	Stem	Crushed	Oral route		127
Campanulaceae	*Monopsis stellarioides*	Herb	Leaves		Oral route		109
Capparaceae	*Boscia coriacea*	Shrub	Fruit; leaves		Oral route; local application		95
Capparaceae	*Cadaba glandulosa*	Shrub	Root; leaves		Oral and nasal routes; local application		95
Capparaceae	*Cadaba rotundifolia*	Shrub	Leaves		Local application		95
Capparaceae	*Maerua pseudopetalosa*	Tree	Root	Cutted and mixed with water	Oral route	Used for snake and scorpion envenoming; also used as repellent	128
Caricaceae	*Carica papaya*	Tree	Seeds	Crushed and boiled with coffee	Oral route	Associated with honey	126
Caryophyllaceae	*Silene macrosolen*	Herb	Root	Fumigation		Used as snake repellent	129
Caryophyllaceae	*Silene macrosolen*	Herb	Root	Fumigation		Used as snake repellent	98
Caryophyllaceae	*Silene macrosolen*	Herb	Root	Fumigation	Nasal route		130
Combretaceae	*Terminalia brownii*	Tree	Bark		Oral route; local application		109
Combretaceae	*Terminalia laxiflora*	Tree	Root	No preparation	Local application		54
Convolvulaceae	*Ipomoea kituensis*	Herb	Root	Crushed and mixed with water	Oral route		53

**Family**	**Species**	**Habit**	**Part used**	**Preparation**	**Traditional therapeutic use**	**Comments**	**Reference**
Convolvulaceae	*Seddera hirsuta*	Herb	Leaves	No preparation	Local application		94
Convolvulaceae	*Seddera hirsuta*	Herb	Leaves	Crushed and mixed with water	Oral route; local application		94
Convolvulaceae	*Seddera bagshawei*	Herb	Leaves		Oral and nasal routes; body wash		95
Convolvulaceae	*Seddera hirsuta*	Herb	Stem		Oral route		95
Cucurbitaceae	*Coccinia grandis*	Climber	Root	Crushed to powder	Local application		131
Cucurbitaceae	*Cucumis dipsaceus*	Herb	Root; leaves	Crushed to juice	Oral route; local application		53
Cucurbitaceae	*Cucumis dipsaceus*	Herb	Root				51
Cucurbitaceae	*Cucumis ficifolius*	Climber	Root	Grind and mixed with honey	Oral route	Used for snake and spider bites and scorpion sting	98
Cucurbitaceae	*Lagenaria siceraria*	Climber	Leaves	Crushed	Oral route		132
Cucurbitaceae	*Cucumis dipsaceus*	Herb	Root	Crushed	Oral route		100
Cucurbitaceae	*Cucumis ficifolius*	Climber	Root	Chewed and swallowed	Oral route		50
Cucurbitaceae	*Cucumis ficifolius*	Climber	Root	Crushed and mixed with water	Oral route		133
Cucurbitaceae	*Cucumis ficifolius*	Climber	Root	Chewed and swallowed only the juice	Oral route		96
Cucurbitaceae	*Momordica trifoliolata*	Climber	Leaves	Powdered	Local application		100
Curcurbitaceae	*Cucumis ficifolius*	Climber	Root	Swallowed	Oral route		118
Cyperaceae	*Cyperus* spp.		Root	Crushed and mixed with water	Oral route	Associated with garlic	123
Dioscoreaceae	*Dioscorea praehensilis*	Climber	Seeds	Crushed and mixed with water	Oral route		128
Ebenaceae	*Euclea racemosa*	Shrub	Leaves	Crushed and mixed with water	Oral route	Associated with *Aloe* spp.	97
Ebenaceae	*Euclea racemosa*	Shrub	Root; bark	Crushed and mixed with water	Oral route		98
Ebenaceae	*Euclea racemosa*	Shrub	Root; bark	Crushed and mixed with water	Oral route		99
Euphorbiaceae	*Acalypha* spp.		Leaves	Crushed and mixed with water	Oral and nasal routes		94
Euphorbiaceae	*Acalypha indica*	Herb	Leaves		Oral and nasal routes	Veterinary use	95
Euphorbiaceae	*Acalypha indica*	Herb	Whole plant		Oral and nasal routes; body wash		95
Euphorbiaceae	*Claoxylopsis andapensis*	Shrub	Bark; leaves	Chopped and mixed with water	Oral route		104
Euphorbiaceae	*Croton macrostachyus*	Tree	Leaves	Leaf juice	Local application		129
Euphorbiaceae	*Croton macrostachyus*	Tree	Root	Chewed and swallowed	Oral route		107
Euphorbiaceae	*Croton macrostachyus*	Tree	Root		Oral route		121
Euphorbiaceae	*Croton macrostachyus*	Tree	Leaves		Oral route		46
Euphorbiaceae	*Croton macrostachyus*	Tree	Root	Crushed, mixed with water	Oral route		123

**Family**	**Species**	**Habit**	**Part used**	**Preparation**	**Traditional therapeutic use**	**Comments**	**Reference**
Euphorbiaceae	*Croton macrostachyus*	Tree	Leaves	Leaf juice	Local application		130
Euphorbiaceae	*Phyllanthus ovalifolius*	Shrub	Root	Chewed and swallowed	Oral route		102
Fabaceae	*Acacia abyssinica*	Tree	Root	Crushed	Oral route	Associated with *Nicotiana tabacum*	132
Fabaceae	*Acacia brevispica*	Tree	Root; bark	Crushed	Oral route	Associated with *Guizotia scabra, Ficus vasta* and *Nicotiana tabacum*	48
Fabaceae	*Acacia etbaica*	Tree	Root; bark	Chewed and swallowed	Oral route		90
Fabaceae	*Acacia mearnsii*	Tree	Stem; bark	Chewed and swallowed	Oral route		90
Fabaceae	*Acacia mellifera*	Tree	Root; leaves	Crushed and mixed with water	Oral route		131
Fabaceae	*Acacia nilotica*	Tree	Root	Crushed to paste	Oral and nasal route	Veterinary use	108
Fabaceae	*Acacia tortilis*	Shrub	Leaves	Crushed to juice	Oral route; local application		53
Fabaceae	*Calpurnia aurea*	Tree	Leaves	Crushed to juice; decoction	Oral route	Also veterinary use	134
Fabaceae	*Calpurnia aurea*	Tree	Root; stem; leaves	Crushed and mixed with water	Oral route	Veterinary use	91
Fabaceae	*Calpurnia aurea*	Tree	Root	Crushed and mixed with water	Oral route	Veterinary use	103
Fabaceae	*Calpurnia aurea*	Tree	Leaves	Crushed and mixed with water	Body wash	Veterinary use	52
Fabaceae	*Calpurnia aurea*	Tree	Leaves	Mixed with milk			135
Fabaceae	*Delonix elata*	Tree	Root	Chewed and swallowed	Oral route		133
Fabaceae	*Dichrostachys cinerea*	Tree	Bark	Chewed and swallowed	Oral route		136
Fabaceae	*Ficus ovata*	Tree	Bark	Crushed		Associated with *Calotropis procera*	53
Fabaceae	*Indigofera arrecta*	Herb	Leaves	No preparation	Local application		54
Fabaceae	*Indigofera oblongifolia*	Shrub	Root		Oral route; local application	Used for scorpion stings	95
Fabaceae	*Indigofera* spp.		Whole plant	Crushed and mixed with water	Oral route; local application		94
Fabaceae	*Indigofera* spp.		Whole plant	Crushed	Local application		95
Fabaceae	*Lotus corniculatus*	Herb	Root	Powdered and mixed with water	Oral route	Associated with tea	52
Fabaceae	*Pterocarpus lucens*	Tree		Crushed and mixed with water	Oral route		123
Fabaceae	*Senna didymobotrya*	Shrub	Root; leaves	Crushed and mixed with water	Oral route		48
Fabaceae	*Senna italica*	Herb	Leaves	Crushed	Local application		94
Fabaceae	*Senna italica*	Herb	Whole plant	Crushed and mixed with water	Oral route; local application		94
Fabaceae	*Senna obtusifolia*	Shrub	Root	Crushed	Local application		100
Fabaceae	*Senna obtusifolia*	Shrub	Root	Crushed	Local application		136
Fabaceae	*Senna petersiana*	Shrub	Root	Crushed and mixed with water	Oral route		133
Fabaceae	*Senna septemtrionalis*	Shrub	Leaves	Crushed and mixed with water	Oral route		52

Family	Species	Habit	Part used	Preparation	Traditional therapeutic use	Comments	Reference
Fabaceae	*Stylosanthes fruticosa*	Herb	Root		Oral route		109
Fabaceae	*Calpurnia aurea*	Tree	Leaves	Smashed and swallowed	Oral route		120
Fabaceae	*Indigofera arrecta*	Herb	Root	Chewed and swallowed	Oral route		107
Fabaceae	*Indigofera articulata*	Herb	Root		Oral route		95
Fabaceae	*Indigofera brevicalyx*	Herb	Root	Chewed and swallowed	Oral route		93
Fabaceae	*Indigofera costata*	Shrub	Root	Chewed and swallowed	Oral route		137
Fabaceae	*Indigofera spicata*	Herb	Root	Chewed and swallowed	Oral route		93
Fabaceae	*Senna alexandrina*	Shrub	Leaves		Oral and nasal routes; local application		95
Francoaceae	*Bersama abyssinica*	Shrub	Stem bark		Oral route; local application		109
Hypericaceae	*Hypericum quartinianum*	Shrub	Root	Crushed and mixed with water	Oral route	Veterinary use	103
Iridaceae	*Lapeirousia schimperi*	Herb	Root	Root is chewed and spit to the animal's nose	Nasal route	Veterinary use	119
Lamiaceae	*Leonotis ocymifolia*	Shrub	Leaves	Powdered	Oral route		132
Lamiaceae	*Leucas* spp.		Leaves	Crushed and mixed with water	Nasal route		94
Lamiaceae	*Leucas zeylanica*	Herb	Stem; leaves	Burned to ash	Local application		128
Lamiaceae	*Salvia nilotica*	Herb	Root	Crushed and mixed with water	Oral route		53
Lamiaceae	*Leonotis ocymifolia*	Shrub	Root	Crushed in paste	Local application		107
Lamiaceae	*Ocimum spicatum*	Herb	Root		Oral route		95
Lamiaceae	*Plectranthus globosus*	Herb	Leaves	Chopped and mixed with water	Oral route	Associated with Alectra sessiliflora	104
Lamiaceae	*Thymus schimperi*	Herb	Whole plant		Oral route; local application		95
Liliaceae	*Gloriosa superba*	Herb	Root		Oral route		110
Malvaceae	*Abutilon mauritianum*	Shrub	Root	Crushed and boiled	Oral route		110
Malvaceae	*Gossypium herbaceum*	Shrub	Root	Chewed and swallowed	Oral route		118
Malvaceae	*Abutilon bidentatum*	Shrub	Root	Crushed to paste	Local application	Veterinary use	115
Malvaceae	*Gossypium arboretum*	Shrub	Root				138
Malvaceae	*Gossypium barbadense*	Shrub	Root	Chewed and swallowed	Oral route; application on the neck		107
Malvaceae	*Gossypium herbaceum*	Shrub	Root	Chewed and swallowed	Oral route	Non-specific detoxifying action	50
Malvaceae	*Gossypium herbaceum*	Shrub	Root	Chewed and swallowed	Oral route		117
Malvaceae	*Malva verticillata*	Herb	Leaves	Crushed and applied	Local application		57
Meliaceae	*Ekebergia capensis*	Tree	Stem; bark	Crushed and mixed with water	Oral route		90
Melianthaceae	*Bersama abyssinica*	Tree	Buds		Oral route	Associated with *Brucea antidysentrica*	46
Menispermaceae	*Stephania abyssinica*	Climber	Root	Chewed and swallowed	Oral route		112

Family	Species	Habit	Part used	Preparation	Traditional therapeutic use	Comments	Reference
Menispermaceae	*Cocculus pendulus*	Shrub	Root		Oral route		95
Moraceae	*Ficus sycomorus*	Tree	Latex	Powdered	Oral route	Associated with *Acacia abyssinica*	132
Moraceae	*Ficus sycomorus*	Tree	Root; stem; bark	Chewed and swallowed	Oral route	Used for snakebite and scorpion sting	123
Moraceae	*Ficus vasta*	Tree	Latex		Oral route	Associated with *Acacia abyssinica*	132
Moraceae	*Ficus vasta*	Tree	Root	Crushed	Oral route	Associated with *Acacia brevispica* and *Nicotiana tabacum*	48
Moringaceae	*Moringa oleifera*	Tree	Root; bark		Oral route; local application		95
Myrtaceae	*Syzygium guineense*	Tree	Fruit	Crushed	Local application		132
Olacaceae	*Ximenia caffra*	Tree	Leaves	Chewed 7 leaves and swallowed only the juice	Oral route		96
Oleaceae	*Jasminum grandiflorum*	Shrub	Root	Crushed and boiled in water	Oral route		133
Oleaceae	*Jasminum abyssinicum*	Climber	Root	Crushed and mixed with water	Oral route		107
Oleaceae	*Jasminum abyssinicum*	Climber	Leaves	Chewed and swallowed	Oral route		113
Oleaceae	*Olea europaea*	Tree	Leaves	Fumigation	Nasal route		139
Oleaceae	*Olea europaea*	Tree	Stem		Oral route; local application		95
Onagraceae	*Ludwigia abyssinica*	Herb	Root; stem		Local application	Associated with other plants	104
Pedaliaceae	*Sesamum indicum*	Herb	Root	Crushed and mixed with milk	Oral route		53
Penaeaceae	*Olinia rochetiana*	Tree	Latex	Crushed to paste	Local application		140
Phyllanthaceae	*Andrachne aspera*	Herb	Root	Chewed and swallowed	Oral route	Induce vomiting	40
Phyllanthaceae	*Andrachne aspera*	Herb	Root	Chewed and swallowed	Oral route		90
Phyllanthaceae	*Phyllanthus sepialis*	Shrub	Root; stem; leaves	Crushed and mixed with water	Oral route	Veterinary use	91
Phytolaccaceae	*Phytolacca dodecandra*	Climber	Leaves	Squeezed and swallowed	Oral route	Prevention; associated with honey	93
Plantaginaceae	*Plantago lanceolata*	Herb	Seeds	Dry or mixed with water	Oral route		141
Plumbaginaceae	*Plumbago zeylanica*	Shrub	Root bark		Local application		109
Plumbaginaceae	*Plumbago zeylanica*	Shrub	Leaves	Chewed and swallowed	Oral route		118
Plumbaginaceae	*Plumbago auriculata*	Climber	Leaves	Chopped, mixed with water to paste	Body wash	Also veterinary use	104
Plumbaginaceae	*Plumbago zeylanica*	Herb	Root	Crushed in powder	Local application		93
Plumbaginaceae	*Plumbago zeylanica*	Herb	Leaves	Chewed and swallowed	Oral route		117
Poaceae	*Cynodon dactylon*	Herb	Aerial parts	Crushed and mixed with butter	Local application		134
Poaceae	*Cynodon dactylon*	Herb	Aerial parts		Local application	Rubbed to the affected skin	120
Poaceae	*Cynodon dactylon*	Herb	Aerial parts	Chewed and swallowed	Oral route		107

**Family**	**Species**	**Habit**	**Part used**	**Preparation**	**Traditional therapeutic use**	**Comments**	**Reference**
Poaceae	*Cynodon dactylon*	Herb	Stem	Crushed	Local application		132
Poaceae	*Cynodon dactylon*	Herb	Root	Decoction	Oral route		131
Poaceae	*Cynodon dactylon*	Herb	Whole plant		Oral route		46
Poaceae	*Cynodon nlemfuensis*	Herb	Whole plant; leaves	Leaves are pounded and swallowed	Oral route (leaves) and body wash (whole plant)	Associated with *Calpurnia aurea*	120
Poaceae	*Eleusine floccifolia*	Herb	Aerial parts	Crushed to paste	Local application		140
Poaceae	*Hyparrhenia hirta*	Herb	Whole plant	Chopped and mixed with water	Oral route	Also veterinary use	104
Poaceae	*Setaria megaphylla*	Herb	Leaves	Crushed	Nasal route; body wash	Veterinary use	91
Polygalaceae	*Polygala abyssinica*	Herb	Root	Chewed and swallowed	Oral route		90
Polygalaceae	*Polygala abyssinica*	Herb	Root	Chewed and swallowed	Oral route		112
Polygalaceae	*Polygala abyssinica*	Herb	Root	Chewed and swallowed	Oral route		98
Polygalaceae	*Polygala obtusissima*	Herb	Whole plant	Crushed and mixed with water	Oral route; local application		94
Polygalaceae	*Polygala abyssinica*	Herb	Root	Chewed and swallowed	Oral route		50
Polygalaceae	*Polygala sphenoptera*	Herb	Root	Chewed and swallowed	Oral route		93
Polygonaceae	*Rumex nervosus*	Shrub	Leaves	Chewed and swallowed	Oral route		111
Polygonaceae	*Rumex nervosus*	Shrub	Root	Chewed and swallowed	Oral route	Non-specific detoxifying action	50
Primulaceae	*Maesa lanceolata*	Tree	Leaves	Crushed to juice	Local application		131
Primulaceae	*Maesa lanceolata*	Tree	Leaves	Chewed and swallowed	Oral route		113
Proteaceae	*Faurea rochetiana*	Tree	Leaves		Local application		46
Ranunculaceae	*Delphinium dasycaulon*	Herb	Root; leaves	Leaf and root juice	Oral route		90
Ranunculaceae	*Thalictrum rhynchocarpum*	Herb	Root		Oral route		46
Ranunculaceae	*Clematis hirsute*	Climber	Root	Crushed to juice	Oral route		53
Rhamnaceae	*Ziziphus spina-christi*	Tree	Root; leaves	Fumigation		Used as snake repellent	90
Rhamnaceae	*Ziziphus abyssinica*	Shrub		Mixed with water			142
Rhamnaceae	*Ziziphus spina-christi*	Tree	Bark		Oral route		95
Rosaceae	*Prunus persica*	Tree		Crushed and mixed with water	Oral route		97
Rubiaceae	*Pentas schimperi*	Shrub	Root	Chewed and swallowed only the juice	Oral route		96
Rubiaceae	*Pavetta gardeniifolia*	Shrub	Leaves		Oral route		47
Rubiaceae	*Pentas lanceolata*	Herb	Root	Crushed and mixed with water	Oral route	Veterinary use	91
Rubiaceae	*Pentas schimperi*	Shrub		Chewed and swallowed	Oral route		113
Rubiaceae	*Rubia cordifolia*	Climber	Root; leaves	Crushed and mixed with water	Oral route		105; 106
Rutaceae	*Ruta chalepensis*	Shrub	Leaves	Infusion	Oral route	Used for pain relief; associated with *Artemisia absinthium*	34

**Family**	**Species**	**Habit**	**Part used**	**Preparation**	**Traditional therapeutic use**	**Comments**	**Reference**
Rutaceae	*Clausena anisata*	Tree	Leaves		Local application		139
Rutaceae	*Clausena anisata*	Shrub	Leaves	Crushed and mixed with water	Oral route		52
Salicaceae	*Dovyalis caffra*	Shrub	Root	chewed and sprayed	Local application		143
Scrophulariaceae	*Verbascum sinaiticum*	Herb	Root	Chewed and swallowed	Oral route		90
Scrophulariaceae	*Alonsoa acutifolia*	Shrub	Root	Chopped and mixed with water	Oral route	Associated with *Plectranthus glandulosus*; also veterinary use	104
Scrophulariaceae	*Barthlottia madagascariensis*	Herb		Concoction	Oral route		104
Simaroubaceae	*Brucea antidysenterica*	Shrub	Fruit	Mixed with honey	Oral route		135
Smilacaceae	*Smilax aspera*	Climber	Leaves	Crushed and mixed with water	Oral route	Veterinary use	114
Solanaceae	*Nicotiana tabacum*	Shrub		No preparation		Used as repellent	34
Solanaceae	*Datura metel*	Herb	Whole plant		Oral route; local application		109
Solanaceae	*Datura metel*	Herb	Leaves	Crushed to juice	Oral route		144
Solanaceae	*Nicotiana tabacum*	Shrub	Root	Chewed or crushed	Local application		50
Solanaceae	*Nicotiana tabacum*	Shrub	Leaves	Crushed and mixed with water	Oral route; local application		139
Solanaceae	*Nicotiana tabacum*	Shrub	Leaves	Powdered	Oral route		132
Solanaceae	*Nicotiana tabacum*	Shrub	Leaves	Chewed and swallowed	Oral route		126
Solanaceae	*Nicotiana tabacum*	Shrub	Whole plant	Fumigation		Used as repellent	126
Solanaceae	*Nicotiana tabacum*	Shrub	Bark; leaves	Infusion	Oral route		48
Solanaceae	*Nicotiana tabacum*	Shrub	Leaves	Powdered and mixed with water	Oral route; local application		52
Solanaceae	*Solanum incanum*	Shrub	Fruit		Oral route	Veterinary use	120
Solanaceae	*Solanum incanum*	Shrub	Fruit; leaves	Rubbed	Local application		143
Solanaceae	*Solanum incanum*	Shrub	Fruit		Local application		133
Solanaceae	*Solanum incanum*	Shrub	Root	Chewed and swallowed	Oral route		90
Solanaceae	*Solanum incanum*	Shrub	Root	Root is chewed and spit to the animal's nose	Nasal route	Veterinary use	119
Solanaceae	*Solanum incanum*	Shrub	Root		Oral route		47
Solanaceae	*Solanum incanum*	Shrub	Root	No preparation	Oral route		131
Solanaceae	*Solanum incanum*	Shrub	Leaves	Crushed to juice	Oral route		144
Solanaceae	*Solanum jubae*	Shrub	Root; fruit; seeds	Crushed to powder	Local application	Veterinary use	115
Solanaceae	*Solanum marginatum*	Herb	Fruit	No preparation	Oral route	Veterinary use	134
Solanaceae	*Solanum* spp.	Shrub	Root	Chewed and swallowed	Oral route		137
Solanaceae	*Withania somnifera*	Shrub	Root	Chewed and swallowed	Oral route		105; 106
Solanaceae	*Withania somnifera*	Shrub	Root	Crushed and mixed with water	Oral route		53
Sterculiaceae	*Dombeya torrida*	Tree	Bark	Crushed to powder	Local application		140
Sterculiaceae	*Sterculia setigera*	Tree	Root; leaves	Crushed to juice	Oral route; local application		53
Verbenaceae	*Clerodendrum*	Shrub	Stem	Burned	Applied hot		98

Family	Species	Habit	Part used	Preparation	Traditional therapeutic use	Comments	Reference
	myricoides				locally		
Verbenaceae	*Clerodendrum myricoides*	Shrub	Stem	Burned	Applied hot locally		99
Verbenaceae	*Cyclonema myricoides*	Herb	Bark	Crushed to powder			57
Verbenaceae	*Priva adhaerens*	Herb	Root	Decoction	Oral route		131
Verbenaceae	*Verbena officinalis*	Herb	Root	Chewed and swallowed	Oral route		50
Verbenaceae	*Verbena officinalis*	Herb	Root	Chewed and swallowed	Oral route		117
Verbenaceae	*Verbena officinalis*	Herb	Root	Chewed and swallowed	Oral route		118
Verbenaceae	*Verbena officinalis*	Herb	Leaves	Crushed and mixed with water	Oral route	Also veterinary use	104
Vitaceae	*Cyphostemma junceum*	Climber	Root	Powdered	Oral route	Used to prevent snakebite	54
Vitaceae	*Cissus adenaucaulis*	Climber	Root	Crushed to paste	Oral route	Veterinary use	108
Vitaceae	*Cissus aphylla*	Climber	Root	Crushed	Oral route; local application	Veterinary use	133
Vitaceae	*Cissus petiolata*	Climber	Stem; leaves				51
Vitaceae	*Cyphostemma acaulata*	Climber		Mixed with oil and water			145
Vitaceae	*Cyphostemma adenocaule*	Climber	Root	Chewed and swallowed	Oral route	Non-specific detoxifying action	50
Vitaceae	*Cyphostemma adenocaule*	Climber	Leaves	Leave pounded to paste	Body wash	Used to prevent snakebite	140
Vitaceae	*Cyphostemma adenocaule*	Climber	Root	Crushed and filtered	Oral route		98
Vitaceae	*Cyphostemma adenocaule*	Climber	Root	Crushed and filtered	Tied on the body	Used as repellent	98
Vitaceae	*Cyphostemma adenocaule*	Climber	Root	Crushed and filtered	Oral route		99
Vitaceae	*Cyphostemma burgeri*	Herb	Root		Oral route		95
Vitaceae	*Cyphostemma cyphopetalum*	Climber	Root; stem	Chewed and swallowed	Oral route		113
Vitaceae	*Cyphostemma junceum*	Climber	Root	Chewed and swallowed	Oral route		117
Vitaceae	*Cyphostemma junceum*	Climber	Root; bark	Crushed to paste and mixed with honey	Oral route		98
Vitaceae	*Cyphostemma junceum*	Climber	Root; bark	Crushed to paste and mixed with honey	Oral route		99
Vitaceae	*Cyphostemma junceum*	Climber	Root	Chewed and swallowed	Oral route	Also veterinary use (local application)	118
Vitaceae	*Cyphostemma oxyphyllum*	Climber	Root; leaves		Oral route	Associated with honey	98
Vitaceae	*Rhoicissus tridentata*	Climber	Root	Chewed and swallowed	Oral route	Human and veterinary use	50
Zygophyllaceae	*Balanites aegyptiaca*	Tree	Root	Crushed and mixed with water	Oral route		133
Zygophyllaceae	*Balanites aegyptiaca*	Tree	Root	Crushed and mixed with water	Oral route; local application		100
